# Zn^2+^ stimulates salivary secretions via metabotropic zinc receptor ZnR/GPR39 in human salivary gland cells

**DOI:** 10.1038/s41598-019-54173-3

**Published:** 2019-11-27

**Authors:** Yoon-Jung Kim, Youhwa Jo, Yeon-Hee Lee, Kyungpyo Park, Hee-Kyung Park, Se-Young Choi

**Affiliations:** 1 Department of Physiology, Dental Research Institute, Seoul National University School of Dentistry, Seoul, 03080 Republic of Korea; 2 Department of Orofacial Pain and Oral Medicine, Kyung Hee University Dental Hospital, Seoul, 02447 Republic of Korea; 3 Department of Oral Medicine and Oral Diagnosis, Dental Research Institute, Seoul National University School of Dentistry, Seoul, 03080 Republic of Korea

**Keywords:** Cellular neuroscience, Neurophysiology, Salivary gland diseases, Translational research

## Abstract

Zn^2+^ is a divalent cation that is essential for many biological activities, as it influences many ion channels and enzymatic activities. Zn^2+^ can evoke G-protein-coupled receptor signaling via activation of the metabotropic zinc receptor ZnR/GPR39. In spite of evidence suggesting the presence of ZnR/GPR39 in salivary gland cells, there has been no evidence of ZnR/GPR39-mediated modulation of salivary gland function. Here we characterized the role of ZnR/GPR39 in human submandibular gland cells. A 0.25% ZnCl_2_ solution evoked secretion of unstimulated and stimulated whole saliva in humans. We found that ZnR/GPR39 is expressed in human submandibular glands and HSG cells. Zn^2+^ increased cytosolic Ca^2+^ concentration ([Ca^2+^]_i_) in a concentration-dependent manner. Muscarinic antagonist had no effect on Zn^2+^-induced [Ca^2+^]_i_ increase, which was completely blocked by the phospholipase C-β inhibitor. As with muscarinic agonist, Zn^2+^ also induced the translocation of aquaporin-5 (AQP-5) to the plasma membrane, which was drastically decreased in ZnR/GPR39-knockdown cells. These data suggest that the metabotropic Zn^2+^ receptor ZnR/GPR39 can modulate salivary secretion in human submandibular gland cells independent of muscarinic or histamine receptor signaling.

## Introduction

Zn^2+^ is a divalent cation that acts as a cofactor for various enzymes^1^. Zn^2+^, which binds to many proteins and regulates their function, plays an important physiological role in many cells including neurons^2–4^. Extracellular Zn^2+^ modulates cellular functions by regulating channels such as the NMDA receptor, GABA_A_ receptor, and purinoceptor^5^. In addition, Zn^2+^ can stimulate a G-protein-coupled receptor (GPCR) that selectively recognizes Zn^2+^. This metabotropic Zn^2+^ receptor, also known as ZnR/GPR39 is present in hippocampal neurons, keratinocytes, colon epithelial cells, and pancreatic cells^6^. ZnR/GPR39 activates phospholipase C-β (PLC-β) as a Gq-coupled receptor and induces cytosolic Ca^2+^ signaling by forming intracellular IP_3_^7^.

Activity-dependent water secretion is the most important physiological function of the exocrine glands, including the salivary glands and kidneys. Exocrine gland cells utilize GPCRs to accept extracellular signals and regulate trafficking of aquaporin (AQP), a water channel protein. In the kidney, vasopressin receptors in the renal collecting duct cells induce cAMP production, leading to membrane translocation of AQP-2/3^8,9^. In contrast, intracellular Ca^2+^ is a key factor controlling salivary secretion in salivary glands^10–12^. Acetylcholine secreted from the parasympathetic terminals acts on the muscarinic receptors of the plasma membrane in salivary gland cells to induce a PLC-β-dependent [Ca^2+^]_i_ increase^13^. Muscarinic receptors in salivary glands induce Ca^2+^ signaling and salivary secretion in a Gq-coupled GPCR- and PLC-β-dependent manner^14–16^. Because Ca^2+^-mobilizing GPCRs in the salivary gland act as an important salivation control factor, identifying and characterizing novel Ca^2+^-mobilizing GPCRs in salivary gland cells is an important aspect of understanding the regulatory mechanism of salivary secretion.

Interestingly, ZnR/GPR39 is expressed in a human submandibular ductal cell line, HSY cell, leading to a Zn^2+^-induced [Ca^2+^]_i_ increase^17^. The interaction of ZnR/GPR39 with another GPCR, CaSR, has also been identified^18^. However, the roles of Zn^2+^ and ZnR/GPR39 in salivary secretion have not been elucidated. It is interesting to study Zn^2+^-induced salivary secretions clinically, since ZnCl_2_ is commonly used to reduce halitosis^19–21^.

In this study, we report that the whole salivary flow rate under resting and stimulated conditions was increased by 0.25% ZnCl_2_ solution. We also investigated the expression of ZnR/GPR39 in human submandibular gland cells and HSG cells, salivary ZnR/GPR39-mediated Ca^2+^ signaling, and translocation of AQP-5, a major water channel in salivary gland cells.

## Results

### Zn^2+^ increases salivation in humans

**Figure 1:**
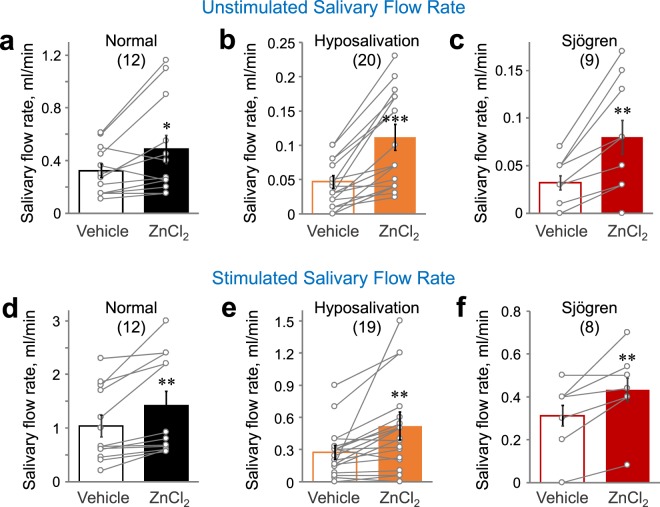
Zn^2+^-containing solution triggers salivation in humans. (**a–c**) Unstimulated/resting salivation was collected from healthy subjects (**a**), patients with hyposalivation (**b**), and Sjögren syndrome patients (**c**) after gargling for 3 min with solutions with or without 0.25% ZnCl2. (**d–f**) Mastication-mediated stimulatory salivation was collected during continuous chewing for 5 min from healthy subjects (**d**), patients with hyposalivation (**e**), and Sjögren syndrome patients (**f**) after 3 min of rinsing with 0.25% ZnCl2 solution. Salivary flow rate was monitored. Numbers of subjects tested were indicated. *P < 0.05, **P < 0.01, ***P < 0.001.

To investigate the effect of Zn^2+^ on salivary secretion, salivary secretion flow rate was measured in human subjects after rinsing with 0.25% ZnCl_2_ solution for 3 min. There was no difference in taste between the vehicle and the ZnCl_2_ solution. Mouth rinsing of ZnCl_2_ solution increased unstimulated salivary secretion in the healthy normal (*P* < 0.05), hyposalivation patients group (*P* < 0.001), and Sjögren syndrome patients (*P* < 0.01) (Fig. 1a–c). In addition, mastication-mediated stimulated salivary secretion was also increased by mouth rinsing with 0.25% ZnCl_2_ solution in the healthy normal (*P* < 0.01), hyposalivation group (*P* < 0.01), and Sjögren syndrome patients (*P* < 0.01) (Fig. 1d–f). The results strongly suggest that 0.25% ZnCl_2_ solution evoked secretion of both unstimulated and stimulated whole saliva in humans.

### GPR39 is expressed in human submandibular glands and HSG cells

**Figure 2:**
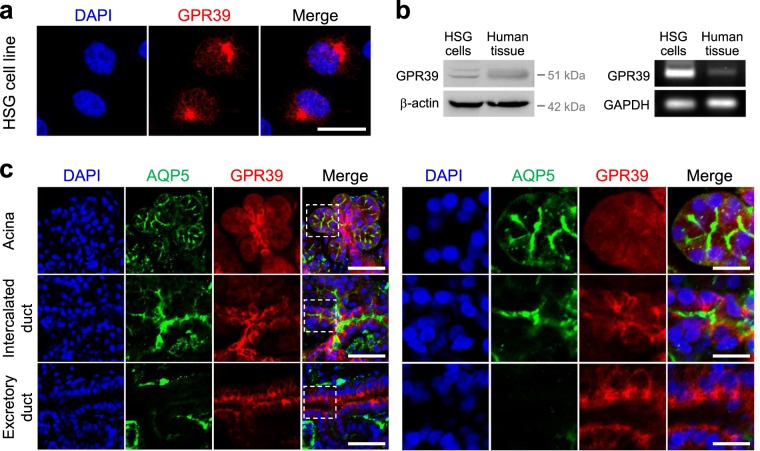
ZnR/GPR39 is expressed in human salivary glands. (**a**) HSG cells were immunostained with anti-human ZnR/GPR39 receptors (red). DAPI (blue) was used as a nuclear marker. Scale bar, 20 μm. (**b**) ZnR/GPR39 expression was confirmed through Western blot and RT-PCR. β-actin and GAPDH were used as loading controls. ZnR/GPR39 protein and mRNA were expressed in a HSG cell line and tissues. (**c**) *left*, Human submandibular gland tissues were immunostained with anti-AQP-5 (green), anti-human ZnR/GPR39 receptors (red), and DAPI (blue). Scale bar, 150 μm. *right*, magnified images of the dotted boxes. Scale bar, 50 μm. ZnR/GPR39 was expressed in the acini and ducts in human salivary gland tissues.

We evaluated the expression of human ZnR/GPR39 in the human submandibular gland cell line HSG (Fig. 2a). Expression of ZnR/GPR39 in human submandibular glands and HSG cells was confirmed by Western blotting and RT-PCR (Fig. 2b). We examined immunohistochemical analysis to observe the distribution of ZnR/GPR39 in human submandibular gland tissue. AQP-5 was used as a marker for the acini and the intercalated duct of salivary gland. Interestingly, ZnR/GPR39 appeared also in acini and intercalated ducts in human submandibular gland (Fig. 2c). We examined the distribution of ZnR/GPR39 in salivary gland tissue compared with the HE image (Fig. S1). The results suggest that ZnR/GPR39 and AQP-5 are expressed in human submandibular glands.

### Zn^2+^ increases [Ca^2+^]_i_ by ZnR/GPR39 activation in HSG cells

**Figure 3:**
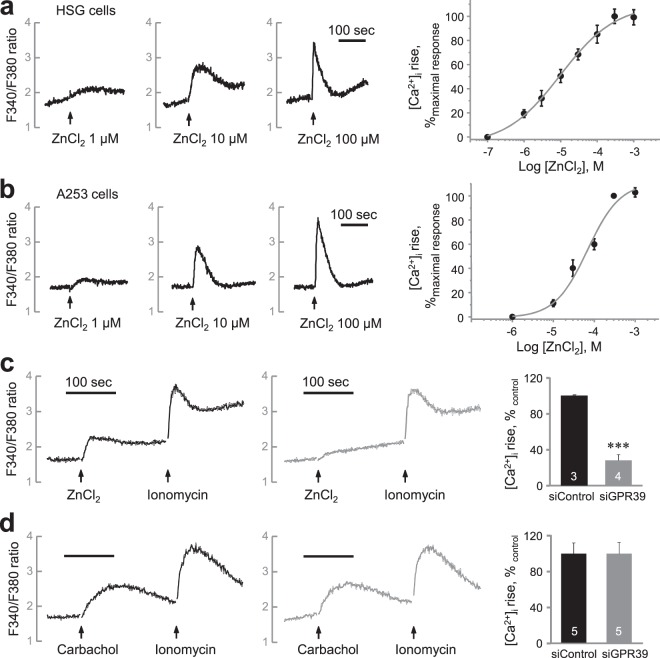
Zn^2+^ evokes [Ca^2+^]_i_ increase via ZnR/GPR39 in human salivary gland cells. (**a,b**) *left*, Fura-2-loaded HSG cells (**a**) and A253 cells (**b**) were challenged with ZnCl_2_ at various concentrations (1, 10, 100 µM) and then monitored for changes in the fluorescence ratio of F340/F380 to represent [Ca^2+^]_i_ level. *right*, Concentration–response relationships were also depicted by measuring the peak height of changes in [Ca^2+^]_i_. A sigmoidal fitting curve is also depicted (gray). (**c,d**) Fura-2-loaded HSG cells transfected with siControl and siGPR39 were challenged with 200 µM ZnCl_2_ (**c**) or 300 µM carbachol (**d**) and then monitored for changes in the fluorescence ratio. Quantification of the change of [Ca^2+^]_i_ mediated by ZnCl_2_ and carbachol was normalized to control groups. The experiments were performed three times independently, and the results were reproducible. ***P < 0.001, compared with control.

**Figure 4:**
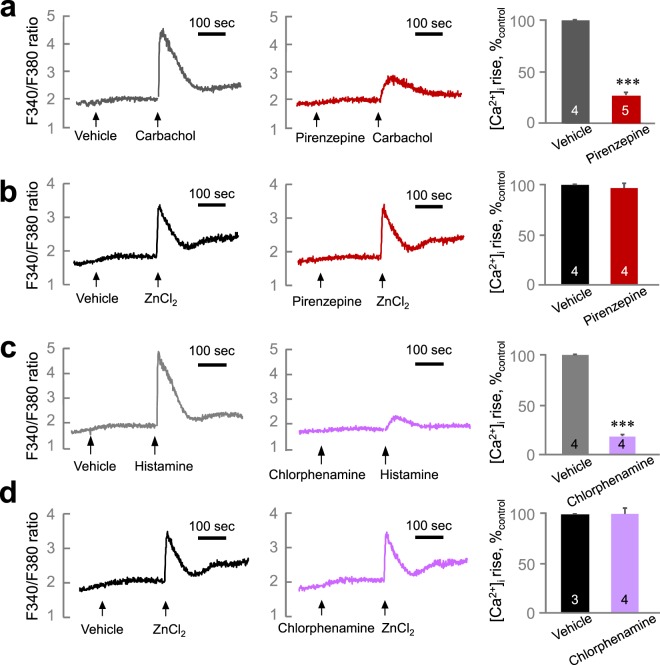
The Zn^2+^-induced [Ca^2+^]_i_ increase is independent from muscarinic or histamine receptor activation. (**a,b**) Fura-2-loaded HSG cells were treated with 300 µM carbachol (**a**) or 30 µM ZnCl_2_ (**b**) after the pre-incubation with 1 µM pirenzepine (right) or vehicle (left). (**c,d**) Cells were treated with 100 µM histamine (**c**) or 30 µM ZnCl_2_ (**d**) with the pre-incubation with 3 µM chlorpheniramine (right) or vehicle (left). Quantification of inhibition on [Ca^2+^]_i_ increase mediated by carbachol or ZnCl_2_ was normalized to vehicle groups. The experiments were performed three times independently, and the results were reproducible. Numbers of data were indicated inside bars. ***P < 0.001, compared with vehicle control.

**Figure 5:**
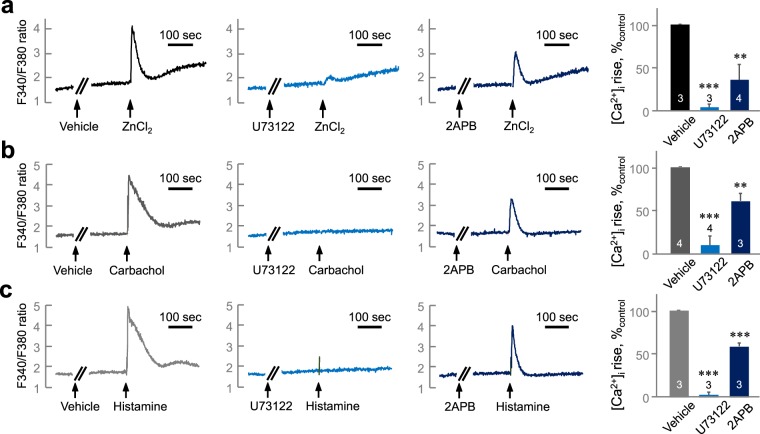
The Zn^2+^-induced [Ca^2+^]_i_ increase is PLC-β-dependent. Fura-2-loaded HSG cells were treated with 30 µM ZnCl_2_ (**a**), 300 µM carbachol (**b**), or 100 µM histamine (**c**) after pre-incubation with vehicle (left), 3 µM U73122 (middle), or 20 µM 2APB (left). Quantification of [Ca^2+^]_i_ increase inhibition mediated by carbachol or ZnCl_2_ was normalized to vehicle groups. The experiments were performed three times independently, and the results were reproducible. Numbers of data were indicated inside bars. **P < 0.01, ***P < 0.001, compared with vehicle control.

We investigated the function of Zn^2+^ in regulating [Ca^2+^]_i_ in salivary gland cells using Fura-2-loaded HSG cells (Fig. 3a). Zn^2+^ increased [Ca^2+^]_i_ in a concentration-dependent manner (EC_50_ = 9.99 ± 2.26 μM). We confirmed the effect of Zn^2+^ on [Ca^2+^]_i_ increase using A253 cells, another cell line that originated from human salivary gland (Fig. 3b). We found Zn^2+^ induced a [Ca^2+^]_i_ increase in a concentration-dependent manner also in A253 cells (EC_50_ = 68.3 ± 13.1 μM). To confirm the involvement of ZnR/GPR39-mediated Ca^2+^ signaling, we prepared the HSG cells which lacks the expression of ZnR/GPR39 by siRNA transfection (Fig. S2). The Zn^2+^-induced [Ca^2+^]_i_ increase was significantly decreased in ZnR/GPR39 knockdown cells compared with the control group (Fig. 3c, *P* < 0.001), whereas carbachol-induced [Ca^2+^]_i_ increase was not altered (Fig. 3d, *P* = 0.997). The results suggest that Zn^2+^ increased [Ca^2+^]_i_ via ZnR/GPR39 activation. Although pirenzepine, a muscarinic M1/M3 receptor antagonist, successfully inhibited the carbachol-induced [Ca^2+^]_i_ increase (Fig. 4a, *P* < 0.001), pirenzepine did not inhibit the Zn^2+^-induced [Ca^2+^]_i_ increase (Fig. 4b, *P* = 0.505). Also chlorpromazine, a histamine H1 receptor antagonist, inhibited the histamine-induced [Ca^2+^]_i_ increase (Fig. 4c, *P* < 0.001), but did not inhibit the Zn^2+^-induced [Ca^2+^]_i_ increase (Fig. 4d, *P* = 0.985). In contrast, pretreatment with U73122 and 2APB commonly inhibited the [Ca^2+^]_i_ increase evoked by Zn^2+^ (Fig. 5a, *P* < 0.01), as well as carbachol (Fig. 5b, *P* < 0.001) and histamine (Fig. 5c, *P* < 0.001). These results suggest that ZnR/GPR39 is present in HSG cells to provide Ca^2+^ signaling via PLC-β activation, which has been reported as a major downstream pathway of ZnR/GPR39 activation^7^.

### ZnR/GPR39 triggers Zn^2+^-induced aquaporin-5 translocation in HSG cells

In salivary gland cells, Ca^2+^ signaling triggers the translocation of AQP-5, causing water secretion. We examined Zn^2+^-induced AQP-5 translocation using an AQP-5 antibody that recognizes the extracellular domains of AQP-5 and a Myc-tag antibody that recognizes the Myc-tagged AQP-5, respectively. Zn^2+^ increased AQP-5 translocation more than four-fold compared to the control group with 15-sec treatment, and did not show significant difference after 3 min (Fig. S3, *P* < 0.001). This result is similar to muscarinic stimulation-mediated AQP-5 translocation in which 15-sec treatment maximizes the amount of AQP-5 in the apical membrane^22^.

**Figure 6:**
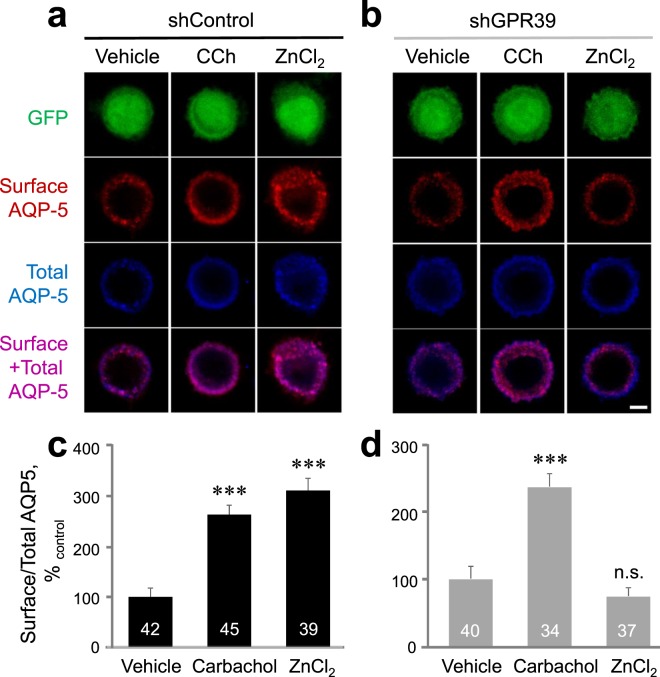
Zn^2+^ successfully increases the surface AQP-5 level on the plasma membrane via ZnR/GPR39 activation. HSG cells cotransfected with shRNA and AQP-5-Myc were treated with vehicle, 300 µM carbachol, or 100 µM ZnCl_2_ for 15 secs (0.25 min) and membrane translocation of AQP-5 was analyzed. (**a,b**) Confocal images of shControl-transfected HSG cells (**a**) and shGPR39-transfected HSG cells (**b**) stained with total AQP-5 (blue) and surface-AQP-5 (red) and expressed with GFP (green). Merged image of total and surface-AQP-5 was depicted (purple). Scale bar, 5 μm. (**c,d**) Quantification of surface-to-total AQP-5 ratio in shControl- (**c**) and shGPR39-transfected HSG cells (**d**). Values are presented as percentages of vehicle. The experiments were performed three times independently, and the results were reproducible. Numbers of counted cells were indicated inside bars. ***P < 0.001, compared with vehicle control. n.s., not significant.

We found that the ratio of surface/total AQP-5 was increased by both Zn^2+^ and carbachol (Fig. 6c, *P* < 0.001) in shControl-transfected cells. However, in ZnR/GPR39 knockdown cells by shGPR39 transfection (Fig. S4), the ratio of surface/total AQP-5 was not increased by Zn^2+^, whereas carbachol did increase the amount of surface AQP-5 (Fig. 6d, *P* < 0.001). These results suggest that Zn^2+^ induces translocation of AQP-5 to the plasma membrane through ZnR/GPR39 activation.

## Discussion

We elucidated Zn^2+^-mediated salivary secretion in humans and identified its cellular and molecular mechanisms. We found that (1) rinsing with 0.25% ZnCl_2_ solution induces secretion of whole saliva, (2) human salivary gland cells express ZnR/GPR39, and (3) Zn^2+^ induces a [Ca^2+^]_i_ increase by activating ZnR/GPR39 and triggers subsequent AQP-5 translocation.

Previous reports showed that the inhibitory effect of Zn^2+^ on halitosis involves two different mechanisms, direct binding with gaseous hydrogen sulfide and inhibitory growth of volatile sulfur compounds^20^. It has also been reported that Zn^2+^ inhibits the growth of oral bacteria^19^. In this report, we observed increased salivary flow rates after rinsing with ZnCl_2_ solution in unstimulated/resting and mastication-stimulated secretions. Interestingly, the increase in salivary flow rate was observed in both healthy and hyposalivation subjects. Thus, considering the high correlation between xerostomia and halitosis^23,24^, our results on Zn^2+^-induced salivary secretion suggest another inhibitory mechanism for halitosis in addition to the previously reported antimicrobial effect. Interestingly, the increase in salivary flow rate was observed in all subjects including those with Sjögren syndrome and those with hyposalivation. This implication that Zn^2+^ could trigger saliva secretion in patients with many different etiological mechanisms, such as impaired muscarinic receptor signaling, increases the clinical potential of our study.

How does a ZnCl_2_ solution rinsing affect salivary acinar cells where producing primary saliva? What absorption mechanism is needed for Zn^2+^-mediated salivation? In our results, ZnR/GPR39 was distributed in the acini and intercalated ducts of the human salivary gland with AQP-5. Expression of ZnR/GPR39 in acini and intercalated ducts serve as evidence for understanding the effect of Zn^2+^ on salivary gland secretion in the oral cavity. We show that exogenous extracellular Zn^2+^ can stimulate GPCRs. Interestingly, 7S-NGF is a Zn^2+^-binding protein in the secretory granules of salivary glands^25^. Thus, it may be possible for Zn^2+^ in secretory granules to be released from salivary gland cells and to act as a messenger in cell-to-cell communication; further investigation on this topic is needed. However, our results suggest that salivary ZnR/GPR39 stimulation occurs at the micromolar level of Zn^2+^, and therefore the intragranular free Zn^2+^ concentration may not be sufficient to activate ZnR/GPR39 in neighboring cells.

What are the characteristics of ZnR/GPR39 signals in human salivary gland cells? We examined whether Zn^2+^ causes muscarinic Ca^2+^ signaling in salivary gland cells. This is important because ZnR/GPR39 regulates the activity of other GPCRs such as CaSR^18^. In our results, the Zn^2+^-mediated [Ca^2+^]_i_ increase was not affected by the muscarinic M1 and M3 antagonist pirenzepine^26^, nor by the histamine H1 antagonist chlorpheniramine^27^, but it has been shown to be blocked by PLC-β inhibitor, U73122 and IP_3_ receptor blocker, 2APB. We also demonstrated that the membrane surface expression of aquaporin-5 is increased by ZnR/GPR39 activity. These results imply that salivary ZnR/GPR39 signaling is independent of both muscarinic and histaminergic signaling, providing cellular and molecular evidence of the mechanism of salivation by Zn^2+^. Previous studies have reported that ZnR/GPR39 is present in HSY cells, a human salivary cell line, to increase intracellular Ca^2+^. However, not all Ca^2+^-mobilizing PLC-β -linked G-protein-coupled receptors commonly cause translocation of aquaporin-5, one of the indicators of salivation. We have studied salivary GPCRs for the past decade. We found that histamine H1 receptor^27^ as well as muscarinic receptors causes aquaporin-5 translocation, but that the stimulation of sphingosine-1-phosphate receptor^28^ and bradykinin B2 receptor^29^ does not affect AQP-5 translocation. Therefore, our current study of salivary secretion by ZnR/GPR39 in human subjects is of significance in salivary translational research. That ZnR/GPR39 acts independently of the muscarinic receptors is also clinically interesting. Our results confirm that Zn^2+^-containing solution increases salivation not only in healthy subjects but also patients with hyposalivation. This indicates the possibility of clinical application of Zn^2+^ for patients with muscarinic dysfunction, especially those with primary Sjögren syndrome-mediated xerostomia, because the secretory dysfunction in primary Sjögren syndrome has been attributed in part to an autoantibody to muscarinic receptors^14,30^.

Taken together, our results suggest that Zn^2+^-containing solutions cause increases in salivary secretion and identified the functional existence and underlying mechanism of salivary ZnR/GPR39, which induces AQP-5 translocation through Ca^2+^ signaling in human salivary gland cells. Our finding that ZnR/GPR39 induces salivary secretion independent of muscarinic signaling may provide therapeutic insight into xerostomia due to muscarinic dysfunction.

## Materials and Methods

### Chemicals

ZnCl_2_, carbachol, histamine, and sulfinpyrazone were purchased from Sigma (St. Louis, MO, USA). Pirenzepine, chlorpheniramine, U73122, and 2APB were obtained from Tocris (Bristol, UK). Thapsigargin was purchased from Alomone Labs (Jerusalem, Israel). Fura-2/acetoxymethyl ester (Fura-2/AM) was obtained from Molecular Probes (Eugene, OR, USA). Fetal bovine serum, Dulbecco’s modified Eagle’s medium (DMEM), and penicillin/streptomycin were purchased from Gibco (Grand Island, NY, USA). Myc-tagged AQP-5 construct was purchased from Origene (Rockville, MD, USA).

### Collection of unstimulated and stimulated whole saliva

Collection of whole saliva was performed using a standardized method. Subjects were instructed to sit upright with the head tilted slightly forward, and a draining method were used^31^. Baseline salivary flow rate was recorded, the subject rested for 5 min, rinsed with 0.25% ZnCl2 solution for 3 min, and salivary flow rate was recorded again for 5 min. Among patients who visited the Department of Oral Medicine, we classified those who had a less than 0.1 ml/min of unstimulated saliva secretion rate as the “hyposalivation group.” Dryness as a subjective symptom was not considered in salivary secretion measurements. These patients reported burning sensation of the tongue, halitosis, burning mouth syndrome, dry mouth, or temporomandibular joint disease. Eight patients were diagnosed with salivary gland inflammation (sialodochitis, atrophy or sialadenitis), 1 patient with dehydration, 2 patients with diabetes mellitus, 2 patients with graft versus host disease, 2 patients with radiation therapy after thyroid cancer, and 9 patients with Sjögren syndrome; in addition, 5 patients were on medication (antipsychotics and antidepressants). Because plasma Zn^2+^ levels were not quantified, it cannot be determined whether those in the hyposalivation group had zinc deficiency. We used water alone as a control, and ZnCl_2_ solution rinsing was used for comparison with the water control. Collection of whole saliva from healthy and hyposalivation subjects was approved by the Institutional Review Board of Seoul National University School of Dentistry (S-D20180019). Thirteen healthy subjects and 29 hyposalivation subjects were included, and informed consent was obtained from all the participants. We also confirm that all methods were performed in accordance with the relevant guidelines and regulations of Seoul National University and Korean government (Ministry of Science and ICT).

### Cell culture and transfection

The HSG and A253 cell lines were maintained in DMEM supplemented with 10% heat-inactivated fetal bovine serum and 1% penicillin/streptomycin. The cell line was cultured in a humidified atmosphere of 95% air + 5% CO_2_. The culture medium was changed every day, and the cell line was subcultured every 3 days. Cells were transfected with DNA constructs and siRNA using Lipofectamine 3000 (Invitrogen) according to the manufacturer’s instructions.

### DNA constructs

shRNA constructs were generated using the FUGW vector under the control of the U6 promoter. The targeted sequences for shRNA were 5’-ACCTTATTCTGGTGTACCTGAT-3’ and 5’-AGCCAGTCCTCTGCAAGGAGAA-3’ for human ZnR/GPR39. siRNAs were Control siRNA (Qiagen cat# SI03650318), GPR39 siRNA (Qiagen cat# SI00430416). We used GFP-expressing shRNA from human ZnR/GPR39 for AQP-5 histology to identify knocked down cells (Fig. S3). For fura-2-based Ca^2+^ measurement, we used siRNA from human ZnR/GPR39, which lacks GFP expression (Fig. S2).

### Measurement of intracellular Ca^2+^ concentration ([Ca^2+^]_i_)

The fluorescent Ca^2+^ indicator Fura-2 was used to determine [Ca^2+^]_i_ level, as previously reported^27,29^. Briefly, cell suspensions were incubated in Locke’s solution (154 mM NaCl, 5.6 mM KCl, 5.6 mM glucose, 2 mM CaCl_2_, 2 mM MgCl_2_, and 5 mM HEPES buffer adjusted to pH 7.4) supplemented with 3 μM Fura-2/AM for 50 min at 37 °C with continuous stirring. Sulfinpyrazone (250 μM) was added to all solutions to prevent Fura-2 leakage. Fluorescence was monitored at 340- and 380-nm dual excitation wavelengths. The ratio of resultant intensities was detected at a 500-nm emission wavelength.

### Immunofluorescent staining

Pieces of human submandibular glands were obtained via radical neck dissection in oral cancer patients who had provided informed consent. The submandibular gland was not infiltrated by malignant cells. The experiments were performed according to the Declaration of Helsinki and the World Medical Association, and were approved by the Institutional Review Board (CRI06002) of Seoul National University Dental Hospital. Human salivary gland tissue was fixed using 4% paraformaldehyde in 0.1 M phosphate-buffered saline (PBS; pH 7.4) at 4 °C overnight. Tissues were cryoprotected in 30% sucrose and serially sectioned to a thickness of 40 μm. HSG cells were fixed in 4% paraformaldehyde in PBS for 30 min at room temperature. After washing with PBS, cells and tissues were blocked with 0.2% Triton X-100 containing 3% bovine serum albumin in PBS for 30 min at room temperature. Cells and tissues were incubated with primary antibodies in blocking buffer overnight at 4 °C and washed three times in PBS. Cells were incubated with secondary antibodies in blocking buffer for 30 min at room temperature. Tissues were incubated with secondary antibodies in blocking buffer for 2 hr at room temperature. The following primary antibodies were used: ab229648 (Abcam, Cambridge, UK) for anti-human ZnR/GPR39 receptors, 2276 (Cell Signaling, Danvers, MA, USA) for anti-Myc, and sc9891 (Santa Cruz Biotechnology, Dallas, TX, USA) for anti-AQP-5 channels. Secondary antibodies were Cy3-conjugated anti-goat secondary and Alexa Flour 488 or 647-conjugated anti-mouse secondary antibody (1:500, Jackson ImmunoResearch Laboratories, West Grove, PA, USA), and Cy3-conjugated goat anti-rabbit IgG (1:500; Invitrogen) antibodies. After mounting, the sections were examined using confocal microscopy with an LSM 700 microscope (Carl Zeiss, Jena, Germany).

### Haematoxylin and Eosin (H&E) staining

The fixed-submandibular gland tissue sectioned at 40 μm thicknesses, and mounted on silane coating-slides. The slides were stained with haematoxylin and eosin dyes using standard procedure and covered with Aqua PolyMount coverslips. After mounting, the sections were examined using digital upright fluorescence microscope (Leica).

### Western blot analysis

Cells and tissues were lysed with RIPA lysis buffer (50 mM Tris-HCl, pH 7.5, 150 mM NaCl, 0.1% sodium dodecyl sulfate, 0.5% sodium deoxycholate, 1% Triton X-100, 2 mM EDTA) containing protease and phosphatase inhibitor cocktail (Roche; Basel, Switzerland) and sonicated. Lysates were centrifuged at 15,000 × g for 15 min at 4 °C, and supernatant was collected. Protein concentration in the supernatant was quantified using the bicinchoninic acid (BCA) protein assay kit (Thermo Scientific, Waltham, MA, USA) according to the manufacturer’s instructions. Lysates were mixed with 5x SDS Laemmli sample buffer (250 mM Tris-HCl, pH 6.8, 10% sodium dodecyl sulfate, 50% glycerol, 5% β-mercaptoethanol, 0.5% bromophenol blue) and then heated for 10 min at 100°C. Proteins were separated by 10% SDS-PAGE and transferred onto a PVDF membrane. After blocking with 5% skim milk, membranes were incubated with primary antibody in 5% BSA TBS-T (20 mM Tris-HCl, pH 7.6, 136 mM NaCl, and 0.1% Tween-20) overnight at 4°C. Membranes were washed with TBS-T and then incubated with horseradish peroxidase (HRP)-conjugated secondary antibodies for 1 h at room temperature. HRP was detected using Super Signal West Femto Maximum Sensitivity substrate (Thermo Scientific, 34096) and a Bio-Image Analyzer (Microchemi/DNR).

### RNA preparation and RT-PCR

Total RNA was isolated from HSG cells and tissues using TRIzol reagent (Life Technologies). For this, 100 ng of total RNA was used for cDNA synthesis using the Super Script III first-strand synthesis system. GAPDH and ZnR/GPR39 were measured using the following PCR cycling protocol: 5 min at 94 °C; 30 cycles of 30 s at 94 °C, 30 s at 63 °C, and 30 s at 72 °C; and a final 10 min at 72 °C. The following primers were used: ZnR/GPR39 sense 5′-GCCACCGGGGTCTCACTTGC-3′ and antisense 5′-GGCCGCAGCCATGATCCTCC-3′; GAPDH sense 5′-CATGAGAAGTATGACAACAGCCT-3′ and antisense 5′-AGTCCTTCCACGATACCAAAGT-3′. The size of the products was 352 bp (GPR39) and 113 bp (GAPDH).

### Quantification of surface AQP-5 channels

For fluorescence-based measurements of the ratio of surface-to-total AQP-5, HSG cells transfected with pCMV6-AQP-5-Myc construct were incubated for 16 hrs. After pretreatment for indicated time with 100 μM ZnCl_2_, cells were fixed in PBS containing 4% formaldehyde and stained for surface AQP-5 using goat anti-AQP-5 antibody (1:100, Santa Cruz) in PBS under a non-permeable condition overnight at 4 °C. Cells were washed three times with PBS, permeabilized in PBS containing 0.5% Triton X-100 for 10 min, and stained for total Myc-tagged AQP-5 using mouse anti-Myc antibody (1:100, Cell Signaling) for 1 hr at room temperature and then a Cy3-conjugated anti-goat secondary and Alexa Flour 488 or 647-conjugated anti-mouse secondary antibody (1:500, Jackson ImmunoResearch Laboratories) for 30 min. Images were acquired with an LSM 700 laser-scanning confocal microscope (Carl Zeiss) using a C-Apo 40 × 1.20 W objective lens. Cells were outlined, and mean fluorescence intensity was measured for each channel using ZEN imaging software (Carl Zeiss). For quantification of surface/total AQP-5 level, the fluorescence intensity of surface AQP-5 was divided by that of total AQP-5. The ratio of surface-to-total AQP-5 fluorescence intensity was compared with that of vehicle-treated controls

### Statistical analysis

Data were analyzed using SPSS version 23 software (IBM, Armonk, NY, USA). All quantitative data are expressed as mean ± SEM. Statistical analysis employed independent Student’s t-tests for single comparisons or one-way ANOVA followed by Fisher’s least significant difference for multiple comparisons. Salivary secretion data from human subjects were analyzed by paired t-test. Detailed results from statistical analysis are shown in Table S1.

## Supplementary information

Supplementary information

## References

[1] Vallee BL, Falchuk KH (1993). The biochemical basis of zinc physiology. Physiol Rev.

[2] Sekler I, Silverman WF (2012). Zinc homeostasis and signaling in glia. Glia.

[3] Sensi SL (2011). The neurophysiology and pathology of brain zinc. J Neurosci.

[4] Sekler I, Sensi SL, Hershfinkel M, Silverman WF (2007). Mechanism and regulation of cellular zinc transport. Mol Med.

[5] Peralta Francisco, Huidobro-Toro Juan (2016). Zinc as Allosteric Ion Channel Modulator: Ionotropic Receptors as Metalloproteins. International Journal of Molecular Sciences.

[6] Hershfinkel Michal (2018). The Zinc Sensing Receptor, ZnR/GPR39, in Health and Disease. International Journal of Molecular Sciences.

[7] Hershfinkel M, Moran A, Grossman N, Sekler I (2001). A zinc-sensing receptor triggers the release of intracellular Ca2+ and regulates ion transport. Proc Natl Acad Sci USA.

[8] Olesen ET, Fenton RA (2017). Aquaporin-2 membrane targeting: still a conundrum. Am J Physiol Renal Physiol.

[9] Valenti G, Procino G, Tamma G, Carmosino M, Svelto M (2005). Minireview: aquaporin 2 trafficking. Endocrinology.

[10] Ambudkar IS (2016). Calcium signalling in salivary gland physiology and dysfunction. J Physiol.

[11] Ambudkar IS, de Souza LB, Ong HL (2017). TRPC1, Orai1, and STIM1 in SOCE: Friends in tight spaces. Cell Calcium.

[12] Nauntofte B (1992). Regulation of electrolyte and fluid secretion in salivary acinar cells. Am J Physiol.

[13] Proctor GB (2016). The physiology of salivary secretion. Periodontol 2000.

[14] Kim N (2015). Effect of Antimuscarinic Autoantibodies in Primary Sjogren’s Syndrome. J Dent Res.

[15] Li J (2006). Effects of pilocarpine on the secretory acinar cells in human submandibular glands. Life Sci.

[16] Kaplan MD, Taylor SE, Ambudkar IS (1994). G-protein- and capacitatively regulated Ca2+ entry pathways are activated by muscarinic receptor stimulation in a human submandibular ductal cell line. Pflugers Arch.

[17] Sharir H, Hershfinkel M (2005). The extracellular zinc-sensing receptor mediates intercellular communication by inducing ATP release. Biochem Biophys Res Commun.

[18] Asraf H (2014). The ZnR/GPR39 interacts with the CaSR to enhance signaling in prostate and salivary epithelia. J Cell Physiol.

[19] Kang JH, Kim DJ, Choi BK, Park JW (2017). Inhibition of malodorous gas formation by oral bacteria with cetylpyridinium and zinc chloride. Arch Oral Biol.

[20] Suzuki N (2018). Two mechanisms of oral malodor inhibition by zinc ions. J Appl Oral Sci.

[21] Tanaka Masami (2002). Secretory Function of the Salivary Gland in Patients with Taste Disorders or Xerostomia: Correlation with Zinc Deficiency. Acta Oto-Laryngologica.

[22] Ishikawa Y, Eguchi T, Skowronski MT, Ishida H (1998). Acetylcholine acts on M3 muscarinic receptors and induces the translocation of aquaporin5 water channel via cytosolic Ca2+ elevation in rat parotid glands. Biochem Biophys Res Commun.

[23] Lee E, Lee YH, Kim W, Kho HS (2014). Self-reported prevalence and severity of xerostomia and its related conditions in individuals attending hospital for general health examinations. Int J Oral Maxillofac Surg.

[24] Rusthen S (2017). Oral disorders, saliva secretion, and oral health-related quality of life in patients with primary Sjogren’s syndrome. Eur J Oral Sci.

[25] Frederickson CJ, Perez-Clausell J, Danscher G (1987). Zinc-containing 7S-NGF complex. Evidence from zinc histochemistry for localization in salivary secretory granules. J Histochem Cytochem.

[26] Rosignoli F, Perez Leiros C (2002). Activation of nitric oxide synthase through muscarinic receptors in rat parotid gland. Eur J Pharmacol.

[27] Kim JH (2009). Histamine H1 receptor induces cytosolic calcium increase and aquaporin translocation in human salivary gland cells. J Pharmacol Exp Ther.

[28] Seo J (2010). Sphingosine-1-phosphate signaling in human submandibular cells. J Dent Res.

[29] Lee K (2017). Human salivary gland cells express bradykinin receptors that modulate the expression of proinflammatory cytokines. Eur J Oral Sci.

[30] Namkoong E, Lee SW, Kim N, Choi Y, Park K (2017). Effect of anti-muscarinic autoantibodies on leukocyte function in Sjogren’s syndrome. Mol Immunol.

[31] Navazesh M, Kumar SK, University of Southern California School of, D. (2008). Measuring salivary flow: challenges and opportunities. J Am Dent Assoc.

