# Supplementary material for: Zn2+ stimulates salivary secretions via metabotropic zinc receptor ZnR/GPR39 in human salivary gland cells
Source: Sci Rep. 2019 Nov 27;9:17648. doi: 10.1038/s41598-019-54173-3 (PMC6881433; doi:10.1038/s41598-019-54173-3)
Supplement: Supplementary file 1 — Supplementary information [file 41598_2019_54173_MOESM1_ESM.pdf]

Supplementary Information for

Zn^{2+} stimulates salivary secretions via metabotropic zinc receptor ZnR/GPR39 in human salivary gland cells

Yoon-Jung Kim[1,*], Youhwa Jo[1,*], Yeon-Hee Lee[2], Kyungpyo Park[1], Hee-Kyung Park[3], and Se-Young Choi[1]

[1] Department of Physiology, Dental Research Institute, Seoul National University School of Dentistry, Seoul 03080, Republic of Korea
[2] Department of Orofacial Pain and Oral Medicine, Kyung Hee University Dental Hospital, Seoul 02447, Republic of Korea
[3] Department of Oral Medicine and Oral Diagnosis, Dental Research Institute, Seoul National University School of Dentistry, Seoul 03080, Republic of Korea

Running title: ZnR/GPR39-mediated human salivary secretion

This PDF file includes:

Figure S1
Figure S2
Figure S3
Figure S4
Figure S5
Figure S6
Figure S7
Table S1

Supplementary Figure and Legends

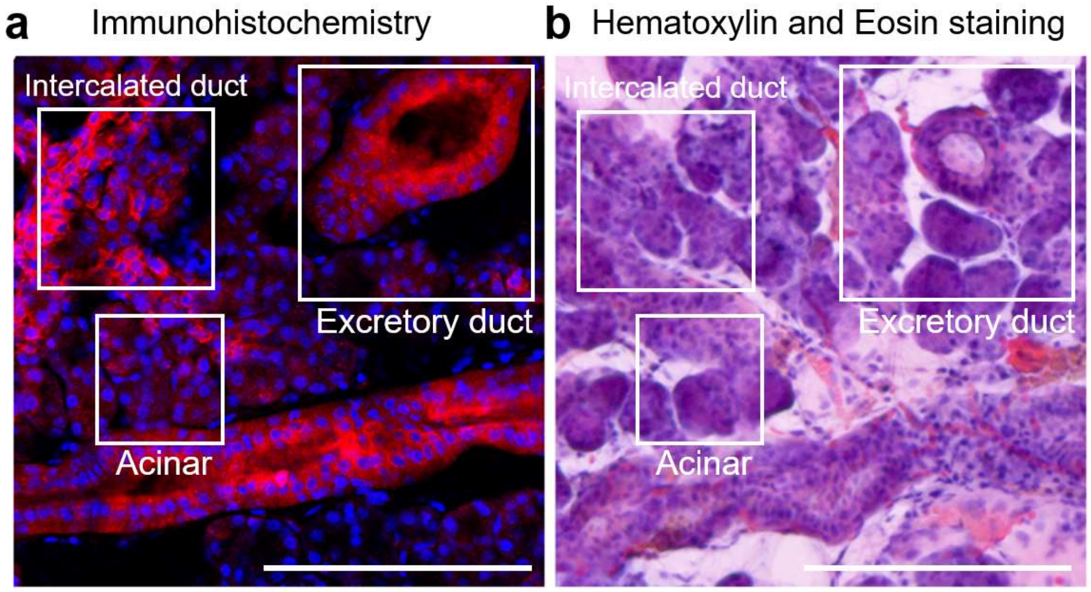

Figure S1. Expression of ZnR/GPR39 in human salivary glands. (**a**) Representative photomicrographs with immunostaining of ZnR/GPR39 (red) in human salivary glands. Scale bar, 150 μm. (**b**) Histology of human salivary glands with Hematoxylin and Eosin staining. Scale bar, 150 μm.

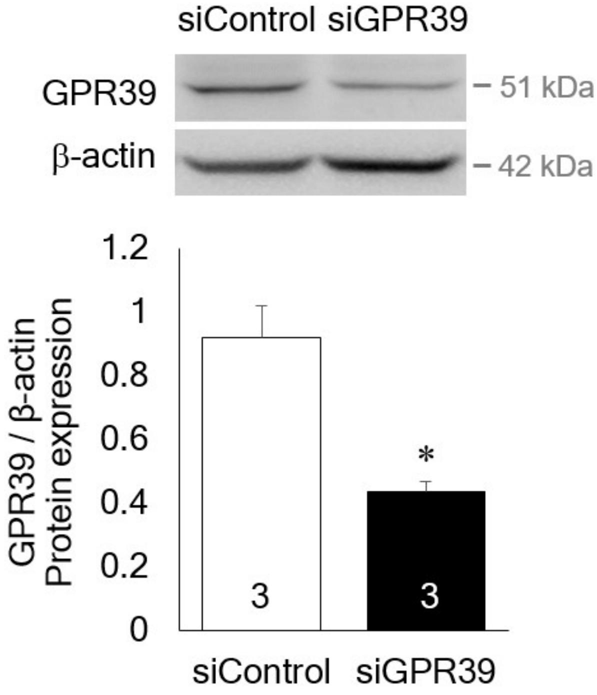

Figure S2. Validation of siRNA knockdown efficiency. HSG cells were transfected with siRNA Control or with siRNA ZnR/GPR39. The protein level of endogenous ZnR/GPR39 by siRNA expression was quantified by Western blot and normalized to that of β-actin (**P* < 0.05; n = 3).

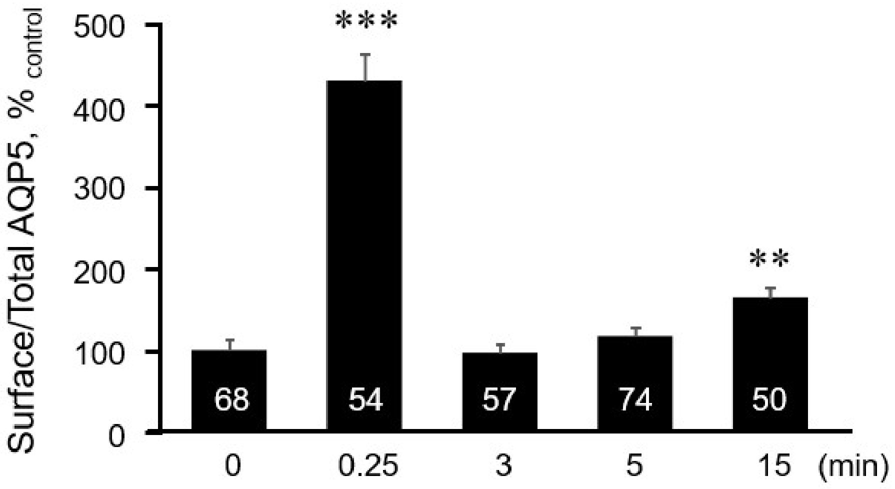

Figure S3. Zn^{2+} successfully increases the surface AQP-5 level on the plasma membrane in HSG cells in a short term. AQP-5-Myc-transfected HSG cells were treated with Zn^{2+} for indicated incubation times (0, 0.25, 3, 5, 15 min), and membrane translocation of AQP-5 was analyzed. Quantification of surface-to-total AQP-5 ratio was depicted as percentages of vehicle treated group. Numbers of data were indicated inside bars. $^{**}P < 0.01$, $^{***}P < 0.001$, compared with vehicle control.

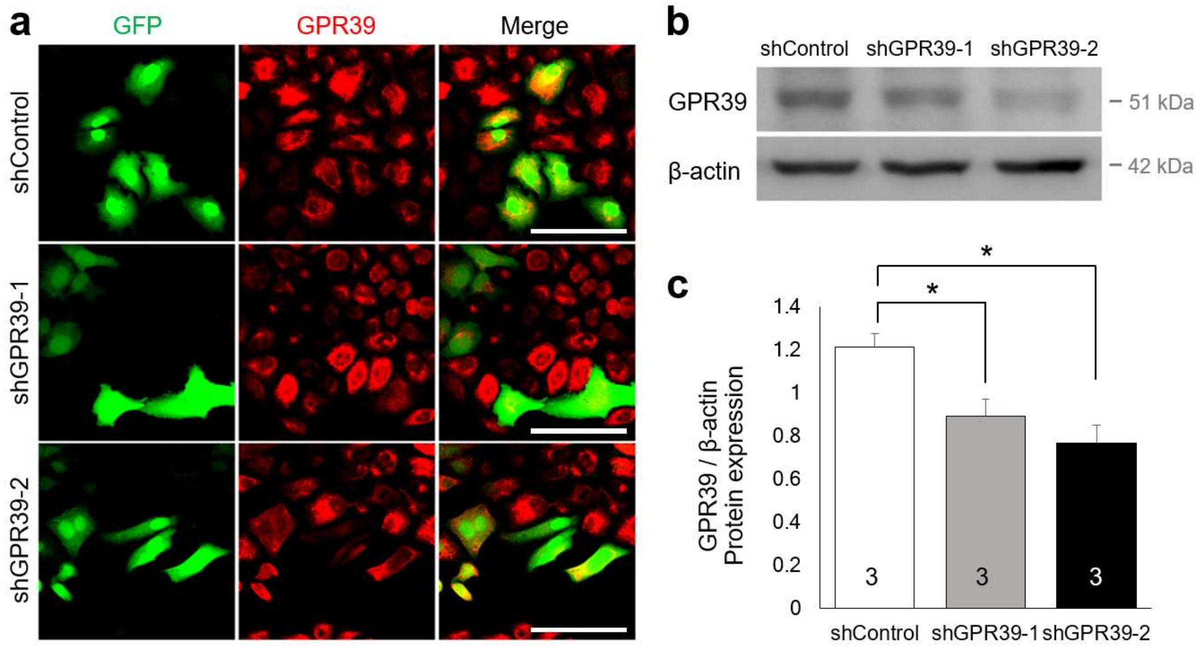

Figure S4. Validation of shRNA knockdown efficiency. HSG cells were transfected with shRNA Control or with shRNA ZnR/GPR39. (**a**) HSG cells were immunostained with anti-human ZnR/GPR39 receptors (red) in shRNA-expressing cells (green). Scale bar: 50 μm. (**b**) Protein level of endogenous ZnR/GPR39 by shRNA expression by Western blot. (**c**) The expression was quantified and normalized to that of β-actin (*$P < 0.05$; n = 3).

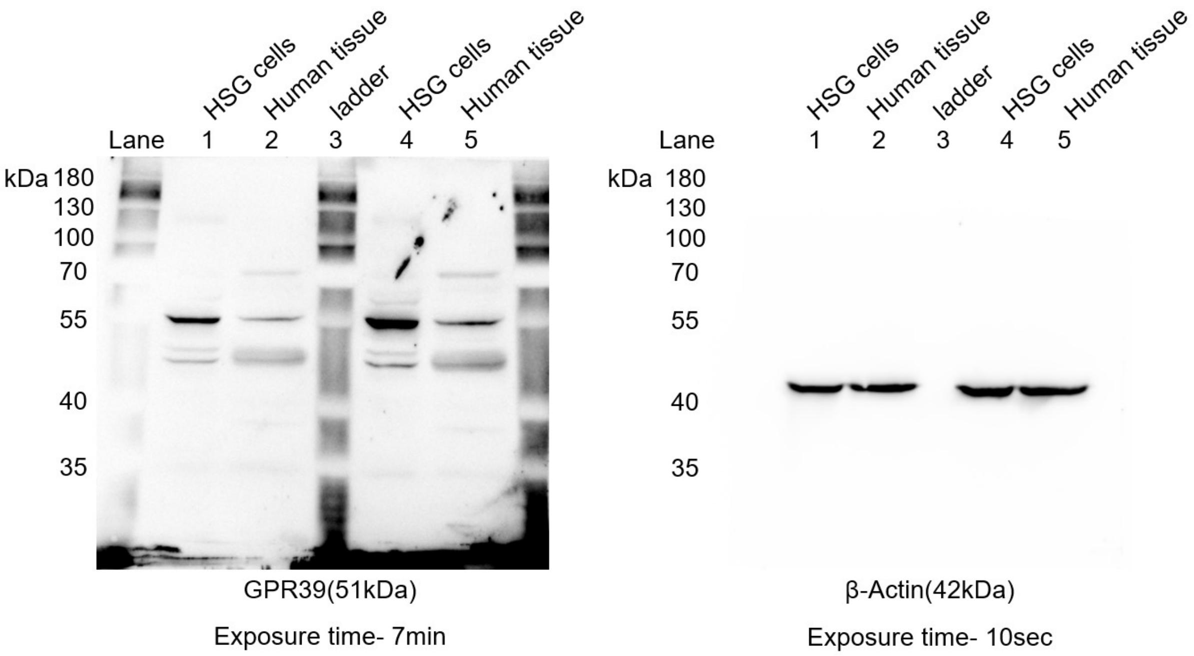

Figure S5. Whole gel blot image for Figure 2b.

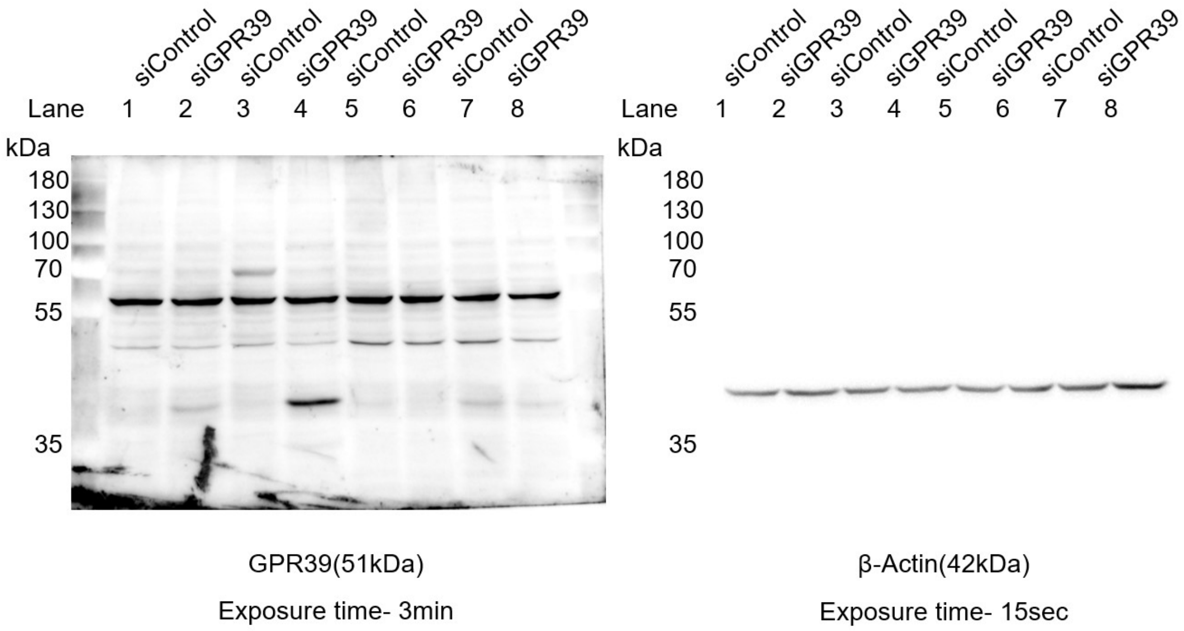

Figure S6. Whole gel blot image for Figure S2.

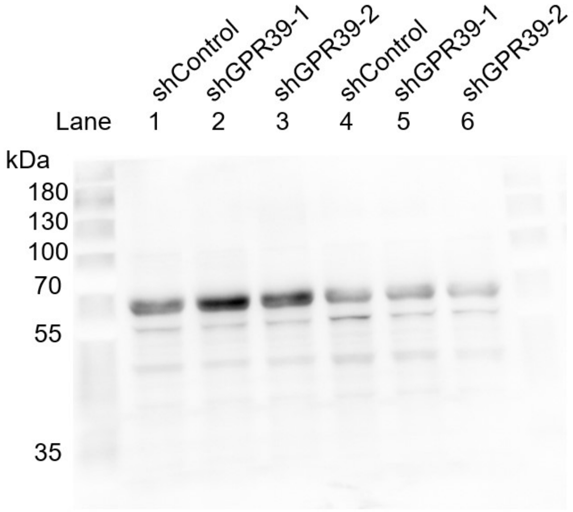

GPR39(51kDa)

Exposure time- 2min

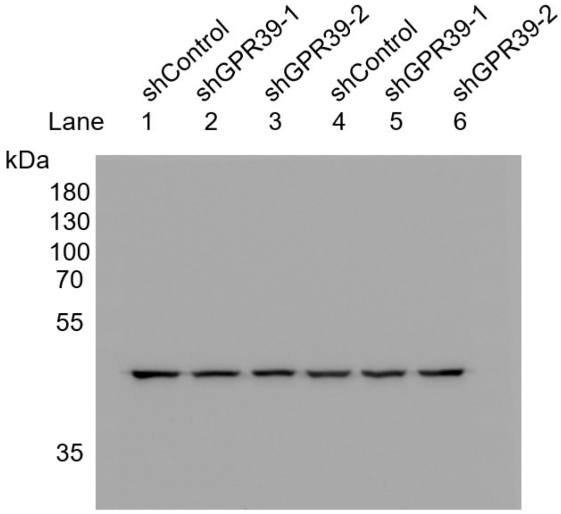

β-Actin(42kDa)

Exposure time- 15sec

Figure S7. Whole gel blot image for Figure S4b.

Table S1. Statistical analysis

Figure number	Test	Subjects	Treatment	Number	Mean	SEM	Statistical test	P value	Degree of freedom	post hoc test	P value
1a	Salivary flow rate	Human : Normal_Unstimulation	Vehicle	12	0.3225	0.052	Paired Student's T-test	0.0308	t(11) = -2.4759		
			ZnCl2	12	0.4858	0.1059					
1b	Salivary flow rate	Human : Hyposalivation_Unstimulation	Vehicle	20	0.0468	0.0073	Paired Student's T-test	0.0000	t(19) = -6.8714		
			ZnCl2	20	0.1117	0.0145					
1c	Salivary flow rate	Human : Sjögren's syndrome_Unstimulation	Vehicle	9	0.0322	0.0083	Paired Student's T-test	0.0047	t(8) = -3.8732		
			ZnCl2	9	0.08	0.0196					
1d	Salivary flow rate	Human : Normal_Stimulation	Vehicle	12	1.0383	0.2024	Paired Student's T-test	0.0058	t(11) = -3.4117		
			ZnCl2	12	1.4183	0.268					
1e	Salivary flow rate	Human : Hyposalivation_Stimulation	Vehicle	19	0.2858	0.0493	Paired Student's T-test	0.0040	t(18) = -3.2939		
			ZnCl2	19	0.52	0.0912					
1f	Salivary flow rate	Human : Sjögren's syndrome_Stimulation	Vehicle	8	0.3125	0.0549	Paired Student's T-test	0.0088	t(7) = -3.5949		
			ZnCl2	8	0.4325	0.062					
3c	[Ca2+]i rise	HSG cells w/ siControl transfection	ZnCl2	3	100	0	Unpaired Student's T-test	0.0000	t(5) = 11.531		
		HSG cells w/ siGPR39 transfection	ZnCl2	4	28.0415	5.2739					
3d	[Ca2+]i rise	HSG cells w/ siControl transfection	Carbachol	5	100	11.9305	Unpaired Student's T-test	0.9972	t(8) = 0.004		
		HSG cells w/ siGPR39 transfection	Carbachol	5	100.0635	12.596					
4a	[Ca2+]i rise	HSG cells	Vehicle - Carbachol	4	100	0	Unpaired Student's T-test	0.0000	t(7) = 23.4179		
			Pirenzepine - Carbachol	5	26.9989	2.7492					
4b	[Ca2+]i rise	HSG cells	Vehicle - ZnCl2	4	100	0	Unpaired Student's T-test	0.5047	t(6) = 0.7093		
			Pirenzepine - ZnCl2	4	96.4421	5.0157					
4c	[Ca2+]i rise	HSG cells	Vehicle - Histamine	4	100	0	Unpaired Student's T-test	0.0000	t(6) = 51.1979		
			Chlorpheniramine - Histamine	4	18.3037	1.5957					
4d	[Ca2+]i rise	HSG cells	Vehicle - ZnCl2	3	100	0	Unpaired Student's T-test	0.9849	t(5) = 0.0199		
			Chlorpheniramine - ZnCl2	4	100.1462	6.2006					
5a	[Ca2+]i rise	HSG cells	Vehicle - ZnCl2	3	100	0	ANOVA	0.0015	F(2,7) = 18.7441	Fisher's test	
			U73122 - ZnCl2	3	3.3386	3.3386					0.0005
			2APB - ZnCl2	4	43.0372	14.6728					0.0064
5b	[Ca2+]i rise	HSG cells	Vehicle - Carbachol	4	100	0	ANOVA	0.0000	F(2,8) = 39.1687	Fisher's test	
			U73122 - Carbachol	4	10.116	10.116					0.0000
			2APB - Carbachol	3	60.9155	8.4634					0.0074
5c	[Ca2+]i rise	HSG cells	Vehicle - Histamine	3	100	0	ANOVA	0.0000	F(2,6) = 329.9546	Fisher's test	
			U73122 - Histamine	3	2.7847	2.7847					0.0000
			2APB - Histamine	3	58.2267	3.7241					0.0000
6c	Surface/Total AQP5	HSG cells w/ shControl transfection	Vehicle	42	100	17.3175	ANOVA	0.0000	F(2,123) = 30.5924	Fisher's test	
			Carbachol	45	262.4289	18.3719					0.0000
			ZnCl2	39	310.4228	23.701					0.0000
6d	Surface/Total AQP5	HSG cells w/ shGPR39 transfection	Vehicle	40	100	18.1825	ANOVA	0.0000	F(2,108) = 25.6546	Fisher's test	
			Carbachol	34	236.4528	19.3628					0.0000
			ZnCl2	37	74.2796	11.9355					0.2719
S2	GRP39 / β-actin ratio	HSG cells	siControl	3	0.9189	0.1027	Unpaired Student's T-test	0.0107	t(4) = 4.5097		
			siGPR39	3	0.4355	0.0307					
S3	Surface/Total AQP5	HSG cells	ZnCl2 untreated (0 min)	68	100	12.2781	ANOVA	0.0000	F(4,298) = 64.5034	Fisher's test	
			ZnCl2 treated for 0.25 min	54	429.2158	33.3261					0.0000
			ZnCl2 treated for 3 min	57	96.4951	11.1175					0.8824
			ZnCl2 treated for 5 min	74	116.6295	10.6216					0.4531
			ZnCl2 treated for 15 min	50	164.2087	11.1529					0.0094
S4c	GRP39 / β-actin ratio	HSG cells	shControl	3	1.2148	0.0626	Unpaired Student's T-test	0.0319<br>0.0123	t(4) = 3.2335<br>t(4) = 4.331		
			shGPR39-1	3	0.8939	0.1333					
			shGPR39-2	3	0.7686	0.0818					
